# Comparative effectiveness of moderate hypofractionation with volumetric modulated arc therapy versus conventional 3D-radiotherapy after radical prostatectomy

**DOI:** 10.1007/s00066-022-01909-2

**Published:** 2022-03-13

**Authors:** Matthias Moll, David D’Andrea, Alexandru Zaharie, Bernhard Grubmüller, Christopher Paschen, Sonja Zehetmayer, Shahrokh F. Shariat, Joachim Widder, Gregor Goldner

**Affiliations:** 1 Department of Radiation Oncology, Comprehensive Cancer Center, Medical University of Vienna, Währinger Gürtel 18–20, 1090 Vienna, Austria; 2 Department of Urology, Comprehensive Cancer Center, Medical University of Vienna, Vienna, Austria; 3 Division of Nephrology and Dialysis, Department of Internal Medicine III, Medical University of Vienna, Vienna, Austria; 4 Center for Medical Statistics, Informatics, and Intelligent Systems, Section for Medical Statistics, Medical University of Vienna, Vienna, Austria; 5 Departments of Urology, Weill Cornell Medical College, New York, NY USA; 6 Department of Urology, University of Texas Southwestern, Dallas, TX USA; 7 Department of Urology, Second Faculty of Medicine, Charles University, Prague, Czech Republic; 8 Institute for Urology and Reproductive Health, I.M. Sechenov First Moscow State Medical University, Moscow, Russian Federation; 9 Division of Urology, Department of Special Surgery, Jordan University Hospital, The University of Jordan, Amman, Jordan

**Keywords:** bNED, Biochemical control, Gastrointestinal toxicity, Genitourinary toxicity, Postoperative

## Abstract

**Purpose:**

Hypofractionated radiotherapy for prostate cancer is well established for definitive treatment, but not well defined in the postoperative setting. The purpose of this analysis was to assess oncologic outcomes and toxicity in a large cohort of patients treated with conventionally fractionated three-dimensional (3D) conformal radiotherapy (CF) and hypofractionated volumetric modulated arc therapy (HF) after radical prostatectomy.

**Methods:**

Between 1994 and 2019, a total of 855 patients with prostate carcinoma were treated by postoperative radiotherapy using CF (total dose 65–72 Gy, single fraction 1.8–2 Gy) in 572 patients and HF (total dose 62.5–63.75 Gy, single fraction 2.5–2.55 Gy) in 283 patients. The association of treatment modality with biochemical control, overall survival (OS), and gastrointestinal (GI) and genitourinary (GU) toxicity was assessed using logistic and Cox regression analysis.

**Results:**

There was no difference between the two modalities regarding biochemical control rates (77% versus 81%, respectively, for HF and CF at 24 months and 58% and 64% at 60 months; *p* = 0.20). OS estimates after 5 years: 95% versus 93% (*p* = 0.72). Patients undergoing HF had less frequent grade 2 or higher acute GI or GU side effects (*p* = 0.03 and *p* = 0.005, respectively). There were no differences in late GI side effects between modalities (hazard ratio 0.99). Median follow-up was 23 months for HF and 72 months for CF (*p* < 0.001).

**Conclusion:**

For radiation therapy of resected prostate cancer, our analysis of this largest single-centre cohort (*n* = 283) treated with hypofractionation with advanced treatment techniques compared with conventional fractionation did not yield different outcomes in terms of biochemical control and toxicities. Prospective investigating of HF is merited.

## Introduction

Radical prostatectomy with or without lymphadenectomy is a widely used therapeutic option for localized prostate cancer (PCA) [1, 2]. Despite definitive treatment, about 30% of patients experience biochemical recurrence [3]. External beam radiotherapy has shown a significant oncologic advantage in terms of reducing these recurrences in both the adjuvant and salvage settings [1, 4, 5].

Hypofractionation (HF) provides a logistic advantage of shorter overall treatment time, allowing more patients to be treated with given resources. This also shortens waiting times, benefitting patients and providers alike, in addition to lowering costs per treatment [6].

In a primary setting, HF for PCA is already well established, both in terms of oncological efficacy and safety [7, 8]. In the postoperative setting, however, no large randomized prospective studies on postoperative HF have been published yet. Some retrospective studies on postoperative HF have been published [9–15], but have included only small cohorts.

In 2013, our department was facing steadily increasing numbers of patients with limited resources. Therefore, we implemented a moderately hypofractionated schedule with a total of 62.5 Gy in 25 fractions instead of a 66 Gy total dose in 33 fractions, reducing treatment times by 25% and setting us up to treat a larger collective of postoperative prostate cancer patients, while using the same number of linear accelerators. To evaluate and compare this regimen with our prior treatment, we collected data regarding tumour control and side effects.

In 2014, Cozzarini et al. [11] showed in the presently largest available retrospective study (*n* = 1176) that moderate HF, administered with helical tomotherapy (*n* = 247), leads to more frequent severe (G3 or higher) late genitourinary (GU) side effects (18% at 5 years) compared with conventional fractionation (CF) (*n* = 929, 7% at 5 years) in postoperative radiotherapy of prostate cancer. In the absence of prospective evidence from randomized trials, these results may have contributed to the limited enthusiasm for HF in the postoperative setting.

The goal of our retrospective study was therefore to report biochemical control, survival, and acute and late gastrointestinal (GI) and GU toxicity in a large cohort of consecutively treated patients after radical prostatectomy, focusing on differences between CF and the largest hitherto reported HF cohort.

## Materials and methods

The analysis of our patient cohort was accepted by the local ethics committee (EK Nr: 1226/2020).

### Patient population

Patients included were treated between January 1994 and December 2019 at the Department of Radiation Oncology. The following inclusion criteria had to be met:

- Prostate cancer treated with radical prostatectomy (RPE) with or without lymph node dissection (LND),
- pT2‑4 without evidence of lymph node involvement or distant metastases (c/pN0M0),
- Documented preoperative, postoperative, and preirradiation prostate specific antigen (PSA) values and
- Adjuvant or salvage radiotherapy: salvage RT was defined as PSA persisting or rising ≥ 0.2 ng/mL after surgery; patients with preradiation PSA ≤ 0.2 ng/mL were classified as having received adjuvant radiotherapy.

### Interventions

Patients were treated with either conventionally fractionated three-dimensional (3D) conformal radiotherapy or hypofractionated volumetric modulated arc therapy, depending on the year of treatment. The switch between modalities happened near the end of 2013. For both groups, target volume definition was performed using computed tomography (CT) and magnetic resonance imaging (MRI). The target volume of the prostatic region for all patients was defined as described by Bolla et al. as “a volume that included the surgical limits from the seminal vesicles to the apex with a security margin to encompass sub-clinical disease in the periprostatic area” [16]. Due to our long observation range, patients were treated with either a 3D conformal 4‑field box or the Volumetric Intensity Modulated Arc Therapy (VMAT) technique in supine position. Planning target volume (PTV) margins were 7 mm for VMAT and 12 mm for 3D conformal radiotherapy. For 3D conformal radiotherapy, constraints were a coverage of 100% of the prescribed dose to 90% of the PTV. No constraints for organs at risk were assessed at that time. For the VMAT technique, constraints are listed in Appendix 1. Assumed α/β ratios, in coordination with our radiobiology department, were 3 Gy for the rectum [17] and 5 Gy for the bladder. As the bowel bag is treated with 2 Gy per fraction and femoral heads are of low priority in VMAT technique radiotherapy, no α/β ratios were assumed. Patients had to drink 250 mL of water 30 min before treatment and were told not to urinate for 1 h before treatment. Therefore, they were assumed to be treated with a full bladder. In the 3D conformal radiotherapy era, image-guided radiotherapy was performed with daily digitally reconstructed radiography. In the hypofractionated era, image-guided radiotherapy was performed by the use of daily ExacTrac (Brainlab, Munich, Germany) images.

For CF, doses were 65–67 Gy for patients without clinical recurrence and 70–72 Gy for patients with clinical recurrence, both at 1.8–2 Gy per fraction. For moderate HF, prescribed doses were 62.5 Gy with a single dose of 2.5 Gy in the absence of clinical recurrence, and 63.75 Gy with 2.55 Gy per fraction was applied to clinical recurrences. Assuming an α/β value of 3 Gy for prostate cancer, this equals 68.8 Gy EQD_2Gy_ for the single dose of 2.5 Gy and 70.8 Gy for the single dose of 2.55 Gy. If the α/β value is supposed to be 1.5 Gy, EQD_2Gy_ values were 71.4 Gy and 73.8 Gy, respectively. Patients at higher risk for lymph-node involvement received irradiation of the pelvic lymph nodes, in case the formula described by Roach et al. yielded a risk of positive lymph nodes of ≥ 15% [18]. Doses to the lymph nodes varied between 45 and 50 Gy with 1.8 to 2 Gy per fraction. The target volume included the external, internal, and common iliac lymph node stations, up to the aortic bifurcation (usually L4/5). Lymph node staging was performed via either LND or CT scan.

Androgen-deprivation therapy (ADT) was administered at the discretion of the attending urologist.

### Outcomes

The primary end point was biochemical control after radiotherapy. Secondary end points were OS, as well as genitourinary and gastrointestinal toxicity according to the Radiation Therapy Oncology Group (RTOG). PSA levels, as well as GI and GU side effects using RTOG criteria [19], were assessed by the radiation oncologist during routine follow-up. The failure of biochemical control was defined as a rise of PSA levels > 0.2 ng/mL [20]. Patients were followed up immediately after therapy, 3 months after the end of therapy, 1 year after therapy, and annually thereafter. Survival data with the reference date December 31, 2019, were retrieved from the population census (Statistik Austria).

### Statistical analysis

Patient baseline characteristics were compared between HF and CF with absolute and relative frequencies and the Χ^2^ test for qualitative variables, and the mean, standard deviation (SD), median and two-sample t‑test or Wilcoxon test for quantitative variables (Table 1). To assess the influence of baseline characteristics on time to biochemical control as the primary end point, univariate Cox regression analyses were performed and hazard ratios (HR), 95% confidence intervals (CI), and *p*-values were reported. All baseline variables with *p* < 0.05 were further considered in a multivariable model. Predefined subgroup analyses were performed for Roach > 15% and lymph node irradiation, and sensitivity analyses were conducted, censoring all patients with at 4‑year follow-up (due to unequal follow-up times between groups). Further Cox regressions were performed for time to death for overall survival, and time to late maximum GI or GU toxicity (grade 2 or higher considered as event). Logistic regression analyses were computed to investigate the influence of HF versus CF on acute maximum GI or GU toxicity (grade 0–1 versus 2 or more). Due to the exploratory and retrospective character of the study, statistical significance was considered at *p* 0.05, but not in a confirmatory manner, and no adjustment for multiplicity was performed. All analyses were done using the R program (R 4.0.3).

**Table 1:** Clinicopathologic features of 855 consecutive patients treated with conventionally fractionated three-dimensional (3D) conformal radiotherapy (CF) or moderately hypofractionated volumetric modulated arc therapy (HF) after radical prostatectomy

	HF	CF	Total	*p*
*n*	283	572	855	–
**pT category (%)**	–	–	–	0.863
*2*	137 (48.4)	282 (49.3)	419 (49.0)	–
*3*	145 (51.2)	271 (47.4)	416 (48.7)	–
*4*	1 (0.4)	19 (3.3)	20 (2.3)	–
**Gleason score (%) after RPE**	–	–	–	< 0.001
*≤* *6*	52 (18.4)	169 (29.5)	221 (25.8)	–
*7*	138 (48.8)	233 (40.7)	371 (43.4)	–
*8–10*	93 (32.9)	134 (23.4)	227 (26.5)	–
*X* (unkown Gleason score)	0 (0.0)	36 (6.3)	36 (4.2)	–
**iPSA in µg/l (mean (SD), median)**	12.33 (13.79), 8.2	11.78 (11.46), 8.7	11.96 (12.27), 8.4	0.13
**PSA after surgery in µg/l (mean (SD), median)**	0.27 (1.10), 0.02	0.19 (0.56), 0.02	0.22 (0.78), 0.02	0.14
**PSA before RT in µg/l (mean (SD), median)**	0.69 (1.11), 0.3	0.66 (1.11), 0.38	0.67 (1.11), 0.33	0.29
**Inclusion of lymph nodes (%)**	190 (67.1)	192 (33.6)	382 (44.7)	< 0.001
**LND (%)**	215 (76.0)	311 (54.4)	526 (61.5)	< 0.001
**Roach >** **15 (%)**	173 (61.1)	281 (49.1)	454 (53.1)	< 0.001
**ADT (%)**	–	–	–	< 0.001
*No*	242 (85.5)	421 (73.6)	663 (77.5)	–
*Yes*	40 (14.1)	149 (26.0)	189 (22.1)	–
*No data available*	1 (0.4)	2 (0.3)	3 (0.4)	–
**Duration of ADT in months (median)**	12	7	10	0.51
**Age (mean (SD))**	68.39 (7.05)	66.13 (6.61)	66.88 (6.83)	< 0.001
**Indication for RT (%)**	–	–	–	0.13
*Adjuvant*	77 (27.2)	186 (32.5)	263 (30.8)	–
*Salvage*	206 (72.8)	386 (67.5)	592 (69.2)	–

*HF* hypofractionation, *CF* conventional fractionation, *PSA* prostate-specific antigen, *iPSA* initial PSA before RPE, *LND* lymphonodectomy, *ADT* Androgen deprivation therapy, *RT* radiotherapy, *RPE* radical prostatectomy, *Roach >* *15%* Risk of lymph node involvement > 15% according to the formula postulated by M. Roach [18] $Riskof\,\textit{lymph}\,node\,\textit{involvement}=\left(\textit{Gleason}\,\textit{score}-6\right)\cdot 10+\frac{2}{3}iPSA$

## Results

Patient characteristics are shown in Table 1. There was a difference in the administration of ADT as well as a higher percentage of patients with pelvic lymph node irradiation in patients treated with moderate HF compared with patients treated with CF. Patients receiving HF were slightly older (2.3 years) and the time between RPE and RT increased, being 24 months for HF and 13 months for CF (16 months for both groups combined). Median follow-up was 23 months for HF and 72 months for CF (42 months for both groups combined).

**Fig. 1:**
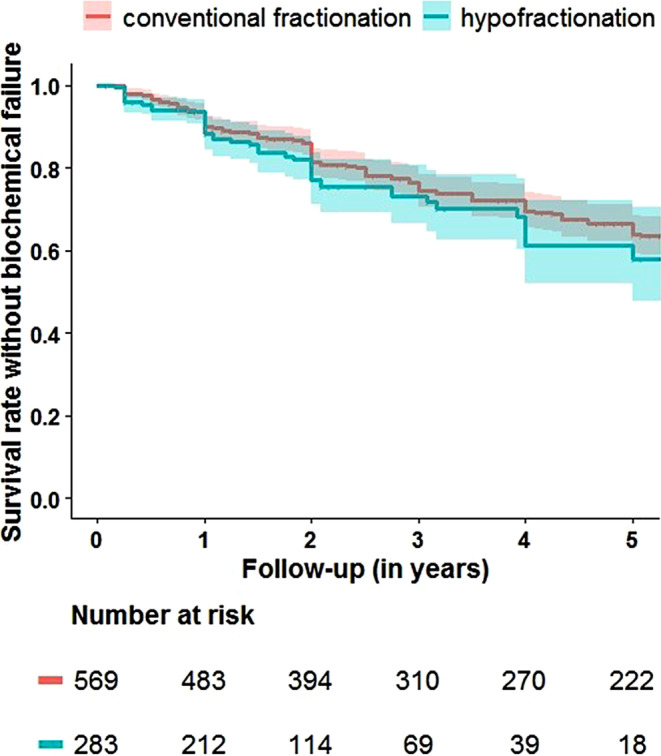
Biochemical control with 95% confidence interval after HF (hypofractionation) and CF (conventional fractionation). *P* = 0.20

The results regarding biochemical control for all patients are displayed in Fig. 1. Biochemical control rates at 2 years were 77% for HF and 81% for CF, and at 5 years were 58% and 64%, respectively (*p* = 0.20). We also performed subgroup sensitivity for lymph node irradiation, as well as Roach > 15% and ≤ 15%. In none of these comparisons were we able to detect any significant difference (*p* = 0.25, 0.11, 0.41, and 0.71, respectively). Due to the relatively shorter follow-up in the HF group, we also performed a sensitivity analysis and censored patients with a follow-up after 48 months; this also revealed no significant differences between the two treatments (4-year biochemical control rates: 61% after HF and 70% after CF, *p* = 0.15).

In a subgroup analysis for adjuvant and salvage indications, no significant influence of fractionation on time to failure of biochemical control was found for both groups (Table 2), but of note, the sample size was smaller with only 8 events (69 censored) in the HF group, compared with 36 events (150 censored) in the CF group. We also performed Kaplan–Meier tests for all patients treated with adjuvant and salvage indications. In the adjuvant group, we observed biochemical control rates for the HF group of 89% and 58% after 2 and 5 years, respectively, with only 5 patients at risk after 5 years, and 92% and 81% for the CF group at 2 and 5 years, respectively. In the salvage group, control rates after 2 and 5 years, respectively, were 73% and 57% in the HF group, and 75% and 54% in the CF group. No significant differences were observed (adjuvant *p* = 0.23 and salvage *p* = 0.68).

**Table 2:** Subgroup analyses for adjuvant and salvage indication regarding time to failure of biochemical control

	Adjuvant	*N* = 263			Salvage	*N* = 589		
	*p*	Hazard ratio (HR)	HR lower CI	HR upper CI	*p*	Hazard ratio (HR)	HR lower CI	HR upper CI
*Hypofractionation (vs CF)*	0.317	1.51	0.67	3.39	0.574	1.10	0.79	1.54
*Age*	0.538	1.02	0.97	1.07	0.201	1.01	0.99	1.04
*pT category (3* *+* *4 vs 2)*	0.002	3.82	1.61	9.05	< 0.001	1.79	1.36	2.36
*Gleason score 7 compared to ≤* *6*	0.389	1.46	0.62	3.47	< 0.001	1.99	1.35	2.92
*Gleason score 8–10 compared to ≤* *6*	0.036	2.46	1.06	5.72	< 0.001	3.04	2.04	4.55
*Staging of lymph nodes via CT (vs surgical)*	0.055	0.54	0.29	1.01	0.28	0.86	0.65	1.13
*PSA before RT*	0.001	1.03	1.01	1.05	0.954	1.00	0.99	1.01
*ADT applied*	0.925	0.97	0.49	1.93	0.707	0.94	0.68	1.30
*Time between RPE and RT*	0.222	1.01	0.99	1.03	0.29	1.00	0.99	1.00
*Inclusion of lymph nodes*	0.205	1.53	0.79	2.94	0.004	1.49	1.13	1.97

*PSA* prostate-specific antigen, *ADT* androgen deprivation therapy, *RT* radiotherapy, *RPE* radical prostatectomy, *HR* hazard ratio, *CI* confidence interval, *CT* computed tomography, *CF* conventional fractionation

Overall survival at 5 years was 93% and 95%, and disease-specific survival at 5 years was 97% and 99%, respectively, for CF and HF. Both differences were not significant (*p* = 0.72 and 0.34).

The highest reported grades of side effects during treatment are displayed in Table 3. Only one patient from the CF group developed a grade 4 late GU toxicity, while none were observed in the HF group. For analytic purposes, we grouped toxicities of grade 0 and 1, and of grade 2 or higher. Acute GI side effects were significantly lower (*p* = 0.02) in patients treated with HF. For acute GU side effects, we also found a significant difference in favour of HF (*p* = 0.03), and a similar result for late GI and GU side effects (*p* = 0.03 and 0.01 in favour of HF, respectively). Overall, the majority of patients showed no or mild side effects, especially regarding late side effects.

**Table 3:** Maximum side effects (Radiation Therapy Oncology Group [RTOG] grading)

		Acute			Late		
	RTOG grade	0–1	2	3	0–1	2	3
*GI*	HF (in %)	75	25	0	92	8	1
	CF (in %)	66	33	1	86	12	2
*GU*	HF (in %)	91	9	0	86	12	2
	CF (in %)	85	14	1	79	15	6

GI gastrointestinal, GU genitourinary, *CF* conventional fractionation, *HF* hypofractionation

Table 4 displays our results regarding the time to onset of late toxicities with an RTOG grade of 2 and higher. The common denominator for late toxicity was any acute toxicity of some kind. For GI toxicity, no further significant parameter was observed and therefore, no multivariable analysis was performed. In terms of late GU toxicity, HF showed higher late GU toxicity only in univariable analysis. Adjusting for age and acute GU toxicity, the difference between HF and CF vanished.

**Table 4:** Univariable Cox regression analysis regarding the time to onset of late gastrointestinal (GI) and uni- and multivariable Cox regression analysis regarding the time to onset of late genitourinary (GU) toxicity of Radiation Therapy Oncology Group (RTOG) grade 2 or higher of factors with possible influence on late toxicity

**GI**	**Univariable Analysis**		**–**	**–**	**–**	**–**	**–**	**–**
*–*	*p*	*HR*	*HR lower CI*	*HR upper CI*	*–*	*–*	*–*	*–*
Age	0.30	1.02	0.99	1.05	–	–	–	–
Hypofractionation (vs CF)	0.98	0.99	0.62	1.59	–	–	–	–
Inclusion of lymph nodes	0.13	0.72	0.48	1.10	–	–	–	–
Time between RPE and RT	0.33	1.00	1.00	1.01	–	–	–	–
Indication for RT salvage (vs adjuvant)	0.37	1.22	0.79	1.88	–	–	–	–
Acute GI side effects grade 2 or higher (vs 0–1)	< 0.001	1.93	1.31	2.86	–	–	–	–
ADT applied	0.51	1.16	0.74	1.82	–	–	–	–
**GU**	**Univariable Analysis**		**–**	**–**	**Multivariable Analysis**		–	–
*–*	*p*	*HR*	*HR lower CI*	*HR upper CI*	*p*	*HR*	*HR lower CI*	*HR upper CI*
Age	< 0.001	1.06	1.03	1.09	< 0.001	1.06	1.03	1.08
Hypofractionation (vs CF)	0.03	1.53	1.03	2.26	0.19	1.32	0.87	1.99
Inclusion of lymph nodes	0.28	1.20	0.87	1.65	–	–	–	–
Time between RPE and RT	0.62	1.00	1.00	1.01	–	–	–	–
Indication for RT salvage (vs adjuvant)	0.68	0.93	0.67	1.30	–	–	–	–
Acute GU side effects grade 2 or higher (vs 0–1)	< 0.001	2.53	1.77	3.61	< 0.001	2.56	1.79	3.65
ADT applied	0.79	1.05	0.73	1.50	–	–	–	–

*CF* conventional fractionation, *HF* hypofractionation, *ADT* androgen deprivation therapy, *RT* radiotherapy, *RPE* radical prostatectomy, *HR* hazard ratio, *CI* confidence interval, *CT* computed tomography, *CF* conventional fractionation

## Discussion

With this study, we want to share our experiences in the treatment of a large postoperative prostate cancer patient cohort with moderate HF. To become a clinically recommendable treatment option, HF should yield equally effective oncological results compared with CF, and side effects during and after treatment should be comparable as well.

As for biochemical control rates, our data show two very similar curves with no significant difference. Our results with rates of 58% for HF and 64% for CF after 5 years are in accord with Tramacere et al. [12] and Kruser et al. [13], who reported rates of 67% for moderate HF after 5 and 4 years in initially 69 and 108 patients, respectively. More recently, these results were confirmed by Franzese et al., who reported rates of 72.3% after 5 years [14]. Macchia et al. reported a rate of 87% at 5 years for moderate HF [15]. However, almost 80% of their reported 124 patient collective received ADT, making a direct comparison with our HF collective, with only 14% receiving ADT, difficult. In summary, neither our results with no significant difference, nor the reported results in the literature with HF biochemical control rates being even higher than those for CF, show an inferiority of HF. However, caution is advised, as our median follow-up for the HF group was only 23 months and our treatment groups were imbalanced regarding ADT prescription, Gleason score, Roach formula results, and radiotherapy indication, favouring the CF group, and lymph node irradiation, favouring the HF group concerning biochemical control rates. However, none of the performed sensitivity analyses changed any of the results. The slightly increased amount of salvage irradiation performed in the HF group might also be an indicator of a worse outcome, as corroborated by several studies [21, 22]. The large difference concerning lymph node irradiation can be explained by a more reluctant practice in pre-VMAT times. Nevertheless, no significant difference between the two treatment groups regarding failure of biochemical control could be detected.

Our results have to be taken with caution, as our HF group was exclusively treated with the VMAT technique and had a 67% inclusion of pelvic lymph nodes, whereas our CF group was treated with 3D conformal radiotherapy and had only 34% inclusion of lymph nodes. In addition, the safety margins were 7 mm for the HF group and 12 mm for the CF group, leading to even more potential toxicity. This was due to the consecutive cohort design of our study, as our department changed not only the dose prescription, but also the irradiation technique at the same time, leading to treatment- and time-disjunct groups.

An increased inclusion of lymph nodes would suggest higher toxicity for HF a priori. However, in terms of acute side effects, we observed significantly lower GI and GU side effects after HF in the VMAT technique compared with CF with a 3D conformal 4‑field-box. This also matches the results of Barra et al. [10], Kruser et al. [13], and the authors of the PRIAMOS‑1 trial [23] who, with the exception of Kruser, also reported low acute toxicity after HF, using Common Terminology for Adverse Events (CTCAE) grading.

For late toxicity, treatment was overall well received, as reported in Table 3. This again is noteworthy, as 67% of the HF patients also received pelvic lymph node irradiation, a known cause of increased late side effects [24, 25]. Regarding the influence of irradiation technique, a British study with over 3000 patients described no significant difference in late GI and GU toxicity between 3D conformal and intensity-modulated radiotherapy [26] after RPE. However, the used doses were low, ranging between 60 and 66 Gy in the CF and 52.5 and 55 Gy in 20 fractions in the HF arm.

Regarding late GU side effects in postoperative HF, it is most important to address Cozzarini et al. [11], who reported a 5-year CTCAE grade 3 GU toxicity rate of 18%, but they administered higher doses compared to the doses administered to our cohort. In addition, improvements in surgery might be reflected in the shift to more postoperative radiotherapy in salvage settings, as indicated in Table 1. Moreover, in the study by Cozzarini et al., the median time between RPE and RT was 5 months in the HF group, whereas our median duration was 24 months.

The most important limitation of our study is its retrospective nature and, more specifically, having observed and compared patient collectives in different periods of time. Moreover, our patient collectives are not balanced, especially regarding inclusion of pelvic lymph nodes, ADT, irradiation technique, Gleason score, and irradiation indication. Moreover, the follow-up in our HF group was relatively short, with a median of 23 months. Due to these differences, we performed various sensitivity subgroup analyses and adjusted analyses where possible to detect any signals of differences in outcomes. However, no such signals could be detected.

As for strengths, to our knowledge this is the largest study on postoperative HF to date, with almost 300 patients included in this treatment group. Our study is monocentric, and a single attending physician has been in charge of treatment, resulting in highly homogeneous treatment delivery and toxicity scoring. However, prospective randomized trials confirming our results regarding HF postoperative radiotherapy are needed.

## Conclusion

Moderate HF might become an option for postoperative prostate cancer patients regarding biochemical control, pending prospective clinical trials. Concerning GI and especially GU toxicities, HF applied with the VMAT technique is well tolerated, and our results challenge those in important prior literature regarding this topic.

## Appendix

**Table 5:** Constraints for organs at risk for hypofractionation

Structure		62.5 Gy/63.75 Gy	62.5/63.75 + 50 Gy
*PTV*	D2%	107% of prescribed dose	107% of prescribed dose
	D50%	Prescribed dose	Prescribed dose
	D98%	95% of prescribed dose	95% of prescribed dose
Rectum	Dmax	66.9 Gy (EQD2 75.9 Gy)	66.9 Gy (EQD2 75.9 Gy)
*α/β 3* *Gy*	D60 Gy (EQD2 64.8 Gy)	20%	20%
	D55 Gy (EQD2 57.2 Gy)	30%	30%
	D46 Gy (EQD2 44.5 Gy)	40%	40%
	D42 Gy (EQD2 39.3 Gy)	45%	45%
	D37 Gy (EQD2 33.2 Gy)	50%	50%
Bladder	Dmax	66.9 Gy (EQD2 73.4 Gy)	66.9 Gy (EQD2 73.4 Gy)
*α/β 5* *Gy*	D60 Gy (EQD2 63.4 Gy)	20%	20%
	D53 Gy (EQD2 53.9 Gy)	40%	40%
	D48 Gy (EQD2 47.5 Gy)	50%	50%
	D43 Gy (EQD2 41.3 Gy)	55%	55%
	D39 Gy (EQD2 36.5 Gy)	60%	60%
	D29 Gy (EQD2 25.5 Gy)	80%	80%
Femoral heads	Dmax	59 Gy	59 Gy
	D50 Gy	5%	5%
Bowel bag	Dmax	–	53 Gy
*Treated with maximally 2* *Gy/fraction*	D48 Gy	–	10%
	D43 Gy	–	15%
	D39 Gy	–	20%

*PTV* Planning target Volume, *DX* volume receiving X Gy, *Gy* gray, *EQD2* equivalent total dose in 2‑Gy fractions
